# Greenshell™ Mussels: A Review of Veterinary Trials and Future Research Directions

**DOI:** 10.3390/vetsci5020036

**Published:** 2018-03-27

**Authors:** Charles T. Eason, Serean L. Adams, Jonathan Puddick, Donato Romanazzi, Matthew R. Miller, Nick King, Sarah Johns, Elizabeth Forbes-Blom, Paul A. Hessian, Lisa K. Stamp, Michael A. Packer

**Affiliations:** 1 Cawthron Institute, Private Bag 2, Nelson 7042, New Zealand; charles.eason@cawthron.org.nz (C.T.E.); serean.adams@cawthron.org.nz (S.L.A.), jonathan.puddick@cawthron.org.nz (J.P.); donato.romanazzi@cawthron.org.nz (D.R.); matt.miller@cawthron.org.nz (M.R.M.); nick.king@cawthron.org.nz (N.K.); sarah.johns@ncg.school.nz (S.J.); 2 Nelson College for Girls, P.O. Box 7060, Nelson 7040, New Zealand; 3 Malaghan Institute of Medical Research, P.O. Box 7060, Wellington 6242, New Zealand; elizabeth.forbesblom@rdls.nestle.com; 4 Department of Medicine, University of Otago, P.O. Box 56, Dunedin 9054, New Zealand; paul.hessian@otago.ac.nz; 5 Department of Medicine, University of Otago, P.O. Box 4345, Christchurch 8140, New Zealand; lisa.stamp@cdhb.health.nz

**Keywords:** anti-inflammation, arthritis, cat, dog, horse

## Abstract

The therapeutic benefits of Greenshell™ mussel (GSM; *Perna canaliculus*) preparations have been studied using *in vitro* test systems, animal models, and human clinical trials focusing mainly on anti-inflammatory and anti-arthritic effects. Activity is thought to be linked to key active ingredients that include omega-3 polyunsaturated fatty acids, a variety of carotenoids and other bioactive compounds. In this paper, we review the studies that have been undertaken in dogs, cats, and horses, and outline new research directions in shellfish breeding and high-value nutrition research programmes targeted at enhancing the efficacy of mussel and algal extracts. The addition of GSM to animal diets has alleviated feline degenerative joint disease and arthritis symptoms, and chronic orthopaedic pain in dogs. In horses, GSM extracts decreased the severity of lameness and joint pain and provided improved joint flexion in limbs with lameness attributed to osteoarthritis. Future research in this area should focus on elucidating the key active ingredients in order to link concentrations of these active ingredients with their pharmacokinetics and therapeutic effects. This would enable consistent and improved efficacy from GSM-based products for the purpose of improved animal health.

## 1. Introduction

The Greenshell™ mussel (GSM; *Perna canaliculus*) is endemic to the coastal waters of New Zealand and has been a part of the staple diet of the indigenous Māori people for hundreds of years [1,2]. Interests in the health benefits of GSM stemmed from the initial observation that coastal Māori communities who consumed GSM had a lower incidence of arthritis than their European or inland counterparts [1,2]. Commercial farming of GSM and research on its potential anti-inflammatory effects began in the early 1970s. Since then, the therapeutic effects have been studied using *in vitro* test systems and animal models, as well as in veterinary and human clinical trials. Over the last 40 years, ca. 150 publications on the therapeutic effects of GSM have been written, however, health claims surrounding GSM products have been embroiled in controversy (e.g., [1,3]). Through *in vitro* experiments and *in vivo* trials, the effects of GSM have been investigated in diseases such as arthritis, cancer, and asthma, as well as on digestive processes (e.g., [1,4,5]). The evidence for anti-cancer properties and activity against some other diseases are not well established [5,6]. There is stronger, although sometimes conflicting, evidence from *in vitro* and *in vivo* trials that extracts of GSM are effective in alleviating the symptoms of inflammation and osteoarthritis [7]. Here, the avoidance of iatrogenic effects encountered with other interventions, such as gastric intolerance of non-steroidal anti-inflammatory drugs (NSAIDS), is an appealing aspect of nutritional supplementation with GSM extracts.

Greenshell mussels contain a range of bioactive lipids; including EPA (eicosapentaenoic acid), DHA (docosahexaenoic acid) and furan fatty acids (F-acids), sphingolipids, phytosterols, diacylglycerols, diterpenes, sesquiterpenes, and saponins, alongside anti-oxidants such as carotenoids, xanthophylls, and anthocyanins [8,9,10]. Most of these compounds are likely biosynthesized in marine algae and then accumulate in the shellfish that feed on them (such as GSM). For some of these bioactives (e.g., EPA and DHA), there is evidence demonstrating effectiveness in relieving the symptoms of arthritis and other inflammatory diseases, not only when they come from GSM but also when they originate from other sources (e.g., see [11,12]). New classes of bioactive compounds found in GSM, such as pro-resolving lipid mediators, are becoming known [13].

*In vitro* studies suggest that the bioactivity observed from extracts of GSM may be through effects on both the cyclo-oxygenase (COX) [8] and lipo-oxygenase (LOX) pathways of arachidonic acid metabolism [2,14,15]. Beneficial activity has been linked to key active ingredients that include the well investigated omega-3 polyunsaturated fatty acids (i.e., EPA and DHA). Other lipids including pro-resolving lipid mediators, bioactive peptides and F-acids may also have a role in the beneficial effects that have been demonstrated and may act via different pathways [13,16].

In the present paper, we review the results from veterinary trials in cats, dogs, and horses where diets were supplemented with GSM. Using this information, we outline promising new research directions which will help to improve future studies in this area and ultimately lead to new strategies to improve animal health and may also impact human health.

## 2. Materials and Methods 

A literature review using the words ‘Greenshell mussel’ and ‘veterinary’ and ‘animal’ was undertaken to identify peer-reviewed publications where GSM preparations were evaluated in veterinary trials. This identified studies using cats, dogs, and horses; more intensive searches using the common name and the systematic names for these animals were then conducted. The key aspects of these trials were tabulated and the results from different trials evaluated. 

## 3. Results

From the literature available, 10 studies on the effects of GSM extracts in dogs, cats, and horses were identified (Table 1). A single study was on cats, seven studies were on dogs, and two studies were on horses. The studies assessed diets supplemented with GSM extracts, usually in powder form. In some of these studies, the effects of GSM extracts alone have been accessed, and in other trials GSM extracts have been combined with other constituents thought to have anti-inflammatory and anti-arthritic effects (see Table 1).

The single clinical trial investigating cats with degenerative joint disease [17] was a blinded randomized controlled trial involving 40 cats (Table 1). A test diet high in EPA and DHA and supplemented with GSM powder, and glucosamine and chondroitin sulphate improved objective measures of mobility when compared to cats on a control diet. Correspondingly, EPA and DHA concentrations in plasma were significantly elevated by the test diet. Because this study evaluated the effects of GSM powder in combination with other supplements, the beneficial effects observed in this study cannot be attributed solely to supplementation with GSM powder.

With one exception [18], the studies conducted on dogs provide evidence on the beneficial effects of GSM extracts for alleviating symptoms of osteoarthritis (Table 1). Whilst there is difficulty directly comparing the results from these studies because of differences in trial design, in the GSM extract used and in the way doses are reported, the majority of the studies reported improvement of symptoms in osteoarthritic dogs supplemented with GSM extracts compared to those with control diets. Two of the studies used objective measures of assessment such as motor activity using ground reaction force measurements [19,20], the remainder of the studies relied on subjective owner assessments and/or semi-objective veterinary assessments.

Two further studies were identified in the literature with horses (Table 1). In a trial with 10 healthy horses in which inflammation was induced via intra-articular injection of interleukin 1 (IL-1), control horses on a normal diet (lacking nutraeutical and ingredients) had increased levels of synovial fluid prostaglandin E2, sulfated glycosaminoglycans, protein concentrations, and leukocyte counts. Horses which were fed 15 mg/kg/day of a dietary nutraceutical containing lipids from GSM, in addition to a normal diet, as well as ingredients including shark cartilage and abalone, had no increase in synovial fluid prostaglandin E2 or sulfated glycosamino glycans [21]. In the second study, a lyophilized GSM powder (Biolane^®^ Vitaco, Auckland, New Zealand) was evaluated in horses with chronic fetlock lameness attributed to osteoarthritis [22]. In this randomised, double-blinded, placebo-controlled study with partial crossover (some horses received placebo then Biolane^®^ following a washout period and vice versa), 20 horses received the placebo (microcrystalline cellulose with non-active colours and flavour to mimic mussel components), and 19 received Biolane^®^ at a dose of 25 mg/kg/day for 48 to 71 days. Clinical evaluations revealed a significant reduction in severity of lameness, improved response to the joint flexion test and reduced joint pain in horses receiving Biolane^®^. 

## 4. Discussion

Our review of the literature has produced insights from trials in cats, dogs, and horses on the value of GSM extracts in veterinary medicine. Multiple research papers report that GSM extracts alleviate arthritis symptoms in animals, although the results from some studies were clearer than in others. Further improvements in effectiveness are likely to be achieved through investigations that provide a better understanding of the active ingredients and the identification of any new unique active ingredients. Moreover, a better understanding of dose response in different species and the optimum duration of treatment when coupled with increased knowledge on mode of action, bioavailability, and pharmacokinetics will further advance this field.

There was difficulty comparing the results of different studies because of the way they were carried out, the exact nature of the GSM extract being evaluated and the fact that some studies combined other supplements with the GSM extract for evaluation [17,21,28]. Nevertheless, the findings from trials in cats, dogs and horses were consistent with indications from pre-clinical studies in rats showing Lyprinol^®^ (a stabilized lipid GSM extract; Pharmalink International, Hong Kong, China) had anti-inflammatory effects and reduced dysmenorrhoea [29] and pain in a rat adjuvant-induced arthritis model [30]. A similar impact on pain was obtained with an alternative solvent extracted lipid-rich fraction of GSM flesh when used in the adjuvant-induced arthritis model in rats [31], while stabilized GSM extracts (CO_2_-supercritical fluid extraction) also had significant anti-inflammatory effects in this model with no apparent adverse effects [32,33]. Heat processing of GSM has been shown to destroy its beneficial activity. The processing of whole GSM and incorporation into foods therefore requires care and suitable processing techniques to avoid destroying any efficacy of the final product [24].

As indicated earlier, experimental and human studies now span some 40 years, and whilst most of the animal veterinary trials have been conducted in the last 15 years there is still considerable inter-study variation in the ingredients and dosages used, making meta-analysis impossible. In the studies assessed during this review, animals demonstrated beneficial effects with GSM doses between 4–49 mg/kg/day, depending on the formulation of the GSM extract. It is noteworthy that in one study 2–8 g/kg/day doses of GSM have been provided to rats with no ill effects [22]. Hence, the margin between an effective dose and a toxic dose of GSM could be substantial and pharmacokinetics of different components of the extracts will be important. This safety data is reassuring if animal feeds are to be supplemented in the future with GSM mussel extracts. 

To better understand the active components of GSM extracts, isolated fractions and new products are being assessed for their anti-inflammatory and anti-allergic effects using *in vitro* and *in vivo* models. Early work establishing the bioactive action of GSM extracts indicated that a fatty acid fraction inhibited COX enzymes involved in the inflammatory response [8] and also inhibited 5-LOX activity, thereby ameliorating the deleterious effect of leukotrienes in arthritis, without gastric side-effects [2,15,34]. Recently, other bioactives including a range of carotenoids (including fucoxanthin), pro-resolving lipids mediators, sulphated polysaccharides, and F-acids have been identified as potential potent anti-inflammatory components of GSM extracts [13,16,35,36]. Although F-acids were first identified in the 1960s and 70s, their beneficial effects are only just being revealed. Using *in vitro* human dendritic cells and immortalised cell (THP-1) models to determine the effects on inflammatory gene regulation and expression allows for the examination of a broad range of potential inflammation-associated genes. Strategies such as this are likely to increase the understanding of the inflammatory pathways that may be impacted by a range of GSM bioactives. The THP-1 cell model has been used successfully in the past to study the impact on gene expression from one particular GSM extract [37]. *In vitro* studies with Perna^®^ GSM extract (FoodScience Corp., Williston, VT, USA) treated, lipopolysaccharide-stimulated THP-1 monocytes demonstrated a decrease in the production of pro-inflammatory cytokines tumor necrosis factor alpha (TNF-α) and interleukin 12p40 (IL-12p40). In addition, a reduction in the reactive oxygen species produced from mitogen-activated rat neutrophils was also established following Perna^®^ GSM extract exposure [37,38]. 

Most of the compounds that are functional or bioactive in the GSM extracts are likely to be biosynthesized in the algae which the shellfish consume. However, it is possible that the algal bioactives may be modified in the shellfish or only be active in the matrix of the shellfish extract. To explore this theory, comparisons between the effects of algal extracts compared to GSM extracts with careful determination of the bioactive content would need to be carried out. Exploration in this area could open up exciting avenues for the use of algal extracts to alleviate similar conditions. As the production of algal cultures allows for increased control of bioactive yield and new strategies to improve production efficacy, this could be an appealing opportunity for future manufacturers of products similar to those tested in the studies assessed here.

As our understanding on the key active ingredients in GSM extracts improves, analytical methodology can be used to assess the concentration of these bioactives in GSM feedstocks, extracts, and final product. This will open up new research avenues as more pharmacokinetic studies become a possibility. It will also allow GSM breeding programmes to select for mussel families that produce higher levels of the bioactives of interest, with good bioavailability yielding a more potent feedstock for therapeutic usage in animals and humans alike.

## 5. Conclusions

In summary, the majority of the animal studies assessed indicated that GSM extracts provided positive effects for the alleviation of mobility-related disorders such as osteoarthritis and degenerative joint disease. When comparing trial outcomes, there were inconsistencies in the degree of benefit provided, which could be attributed to the different dosages used and the different types of GSM extracts used. In turn, these could affect the concentrations of key bioactives present. Further research is needed to clarify the active ingredients in GSM extracts, to elucidate credible biological mechanisms, and to further optimize the concentration of active ingredients to allow continual improvement in the efficacy of GSM extracts versus NSAIDs and other conventional therapies for the treatment of osteoarthritis.

## Figures and Tables

**Table 1:** Table summarizing studies evaluating the effects of Greenshell™ mussel (GSM; *Perna canaliculus*) extracts in cats, dogs, and horses.

Animal	Study Type	*N*	Supplement/Diet	Dosage	Duration (Day)	Measurements	Results	Reference
Cat	Randomized, controlled trial, double blinded, naturally occurring DJD	40	Dry expanded diet incorporating EPA, DHA, GSM extract, and glucosamine/chondroitin sulfate	4 mg/kg/day	70	Subjective owner assessments; semi-objective veterinary assessments; objective active monitoring	Diet supplementation improved objective measures of mobility in cats with DJD associated pain	Lascelles et al. 2010 [17]
Dog	Randomized control trial, double blinded, placebo controlled, naturally occurring DJD	31	Powdered GSM extract was incorporated into test diet	0.3% DM	42	Semi-objective veterinary assessments	Reduced arthritis score, joint pain and joint swelling scores. No change in joint crepitus or range of joint movement	Bui and Bierer 2003 [23]
	Randomized, controlled trial, double blinded, naturally occurring DJD	96	Powdered GSM extract plus standard diet (18–30 mg/kg/day), semi-moist treatment incorporating GSM extract (18–30 mg/kg/day) and dry main meal with GSM (0.3% DM)	18–30 mg/kg/day or 0.3% DM	42	Semi-objective veterinary assessments	Incorporation of GSM mussel extract reduced arthritic score, joint pain and swelling scores irrespective of the experimental diet form provided	Bierer and Bui 2002 [24]
	Randomized, controlled trial, placebo controlled, naturally occurring DJD	70	Powdered GSM extract mixed into the animal’s normal diet (chondrotin sulphate also evaluated)	11 mg/kg/day	84	Subjective owner assessments; semi-objective veterinary assessments	No effect observed	Dobenecker et al. 2002 [18]
	Not randomized controlled trial. Naturally occurring osteoarthritis	85	Dry expanded diet incorporating GSM extract	0.3% DM	50	Semi-objective veterinary assessments	Reduced arthritic score, improved mobility, improved manipulation (pain, swelling, crepitus, movement range)	Servet et al. 2006 [25]
	Double blinded, naturally occurring OA	30	Balanced diet incorporating GSM extract (Medi-Cal/Royal Canin, St. Charles, MO, USA)	Not Stated	90	Subjective owner assessments; objective assessments.	OA symptoms were reduced by balanced diet and further reduced by the inclusion of GSM extract in the diet.	Rialland et al. 2013 [20]
	Randomized, controlled trial, placebo controlled, naturally occurring DJD	81	Oral tablets containing GSM extract	22–37 mg/kg/day	56 *	Subjective owner assessments; semi-objective veterinary assessments	Improved clinical symptoms	Pollard et al. 2006 [26]
	Randomized, double controlled trial, placebo controlled, double blinded, naturally occurring OA	45	Oral capsules containing GSM extract (Lyproflex^®^; VMD, Arendonk, Belgium)	20–49 mg/kg/day	56	Subjective owner assessments; semi-objective veterinary assessments; objective assessments	Chronic orthopedic pain alleviated. However, GSM extract less effective than carprofen	Hielm-Björkman et al. 2013 [27]
Horse	Randomized controlled trial, placebo controlled, induced inflammation	10	Dietary nutraceutical containing GSM extract, shark cartilage, abalone and *Biota orientalis* lipid extract	15 mg/kg/day	29	Semi-objective veterinary assessments; objective assessments	No increase in synovial fluid prostaglandin E2 or sulfated glycosamino glycans in horses fed supplement	Pearson et al. 2009 [21]
	Randomized, controlled trial, double blinded, placebo controlled, partial crossover naturally occurring OA.	30	Supplement containing GSM extract (Biolane^®^) added to feed	25 mg/kg/day	48–71	Semi-objective veterinary assessments	Reduction in severity of lameness, improved response to the joint flexion test, reduced joint pain	Cayzer et al. 2012 [22]

%DM = percentage of dietary material; DHA = docosahexaenoic acid; DJD = degenerative joint disease; EPA = eicosapentaenoic acid; OA = osteoarthritis. * Study was extended an additional 56 days and Greenshell mussel extract fed to all dogs. Results of this extension also indicated benefit. ^†^ An initial trial also summarised in this paper was used to determine no adverse effects of the nutraceutical supplement on otherwise healthy animals.

## References

[1] Cobb C.S., Ernst E. (2006). Systematic review of a marine nutriceutical supplement in clinical trials for arthritis: The effectiveness of the new zealand green-lipped mussel *Perna canaliculus*. Clin. Rheumatol..

[2] Halpern G.M. (2000). Anti-inflammatory effects of a stabilized lipid extract of *Perna canaliculus* (lyprinol). Allerg. Immunol. (Paris).

[3] Doggrell S.A. (2011). Lyprinol—Is it a useful anti-inflammatory agent?. Evid. Based Complement. Alternat. Med..

[4] Stamp L.K., James M.J., Cleland L.G. (2005). Diet and rheumatoid arthritis: A review of the literature. Semin. Arthritis Rheum..

[5] Ulbricht C., Chao W., Costa D., Nguyen Y., Seamon E., Weissner W. (2009). An evidence-based systematic review of green-lipped mussel (*Perna canaliculus*) by the natural standard research collaboration. J. Diet. Suppl..

[6] Torres D.M., Tooley K.L., Butler R.N., Smith C.L., Geier M.S., Howarth G.S. (2008). Lyprinol™ only partially improves indicators of small intestinal integrity in a rat model of 5-fluorouracil-induced mucositis. Cancer Biol. Ther..

[7] Szechinski J., Zawadzki M. (2011). Measurement of pain relief resulting from the administration of *Perna canaliculus* lipid complex pcso-524™ as compared to fish oil for treating patients who suffer from osteoarthritis of knee and/or hip joints. Reumatologia.

[8] McPhee S., Hodges L.D., Wright P.F.A., Wynne P.M., Kalafatis N., Harney D.W., Macrides T.A. (2007). Anti-cyclooxygenase effects of lipid extracts from the new zealand green-lipped mussel, *Perna canaliculus*. Comp. Biochem. Physiol. B Biochem. Mol. Biol..

[9] Taylor A.G., Savage C. (2006). Fatty acid composition of new zealand green-lipped mussels, *Perna canaliculus*: Implications for harvesting for n-3 extracts. Aquaculture.

[10] Miller M.R., Pearce L., Bettjeman B.I. (2014). Detailed distribution of lipids in greenshell mussel (*Perna canaliculus*). Nutrients.

[11] Cleland L.G., James M.J., Proudman S.M. (2006). Fish oil: What the prescriber needs to know. Arthritis Res. Ther..

[12] Ameye L.G., Chee W.S.S. (2006). Osteoarthritis and nutrition. From nutraceuticals to functional foods: A systematic review of the scientific evidence. Arthritis Res. Ther..

[13] Serhan C.N. (2014). Pro-resolving lipid mediators are leads for resolution physiology. Nature.

[14] Whitehouse M.W., Macrides T.A., Kalafatis N., Betts W.H., Haynes D.R., Broadbent J. (1997). Anti-inflammatory activity of a lipid fraction (lyprinol) from the nz green-lipped mussel. Inflammopharmacology.

[15] Treschow A.P., Hodges L.D., Wright P.F.A., Wynne P.M., Kalafatis N., Macrides T.A. (2007). Novel anti-inflammatory omega-3 pufas from the new zealand green-lipped mussel, *Perna canaliculus*. Comp. Biochem. Physiol. B Biochem. Mol. Biol..

[16] Wakimoto T., Kondo H., Nii H., Kimura K., Egami Y., Oka Y., Yoshida M., Kida E., Ye Y., Akahoshi S. (2011). Furan fatty acid as an anti-inflammatory component from the green-lipped mussel *Perna canaliculus*. Proc. Natl. Acad. Sci. USA.

[17] Lascelles B.D.X., DePuy V., Thomson A., Hansen B., Marcellin-Little D.J., Biourge V., Bauer J. (2010). Evaluation of a therapeutic diet for feline degenerative joint disease. J. Vet. Intern. Med..

[18] Dobenecker B., Beetz Y., Kienzle E. (2002). A placebo-controlled double-blind study on the effect of nutraceuticals (chondroitin sulfate and mussel extract) in dogs with joint diseases as perceived by their owners. J. Nutr..

[19] Hielm-Björkman A., Tulamo R.M., Salonen H., Raekallio M. (2007). Evaluating complementary therapies for canine osteoarthritis part I: Green-lipped mussel (*Perna canaliculus*). Evid.-Based Complement. Altern. Med..

[20] Rialland P., Bichot S., Lussier B., Moreau M., Beaudry F., del Castillo J.R., Gauvin D., Troncy E. (2013). Effect of a diet enriched with green-lipped mussel on pain behavior and functioning in dogs with clinical osteoarthritis. Can. J. Vet. Res..

[21] Pearson W., Orth M.W., Lindinger M.I. (2009). Evaluation of inflammatory responses induced via intra-articular injection of interleukin-1 in horses receiving a dietary nutraceutical and assessment of the clinical effects of long-term nutraceutical administration. Am. J. Vet. Res..

[22] Cayzer J., Hedderley D., Gray S. (2012). A randomised, double-blinded, placebo-controlled study on the efficacy of a unique extract of green-lipped mussel (*Perna canaliculus*) in horses with chronic fetlock lameness attributed to osteoarthritis. Equine Vet. J..

[23] Bui L.M., Bierer T.L. (2003). Influence of green lipped mussels (*Perna canaliculus*) in alleviating signs of arthritis in dogs. Vet. Ther..

[24] Bierer T.L., Bui L.M. (2002). Improvement of arthritic signs in dogs fed green-lipped mussel (*Perna canaliculus*). J. Nutr..

[25] Servet E., Biourge V., Marniquet P. (2006). Dietary intervention can improve clinical signs in osteoarthritic dogs. J. Nutr..

[26] Pollard B., Guilford W.G., Ankenbauer-Perkins K.L., Hedderley D. (2006). Clinical efficacy and tolerance of an extract of green-lipped mussel (*Perna canaliculus*) in dogs presumptively diagnosed with degenerative joint disease. N. Z. Vet. J..

[27] Hielm-Bjorkman A., Roine J., Elo K., Lappalainen A., Junnila J., Laitinen-Vapaavuori O. (2012). An un-commissioned randomized, placebo-controlled double-blind study to test the effect of deep sea fish oil as a pain reliever for dogs suffering from canine OA. BMC Vet. Res..

[28] Pearson W., Orth M.W., Karrow N.A., MacLusky N.J., Lindinger M.I. (2007). Anti-inflammatory and chondroprotective effects of nutraceuticals from sasha's blend in a cartilage explant model of inflammation. Mol. Nutr. Food Res..

[29] Shiels I.A., Whitehouse M.W. (2000). Lyprinol: Anti-inflammatory and uterine-relaxant activities in rats, with special reference to a model for dysmenorrhoea. Allerg. Immunol. (Paris).

[30] Lee C.-H., Lum J.H.-K., Ng C.K.-C., McKay J., Butt Y.K.-C., Wong M.-S., Lo S.C.-L. (2009). Pain controlling and cytokine-regulating effects of lyprinol, a lipid extract of *Perna canaliculus*, in a rat adjuvant-induced arthritis model. Evid. Based Complement. Alternat. Med..

[31] McPhee S., Hodges L.D., Wright P.F.A., Wynne P.M., Kalafatis N., Macrides T.A. (2010). Prophylactic and therapeutic effects of *mytilus edulis* fatty acids on adjuvant-induced arthritis in male wistar rats. Prostaglandins Leukot. Essent. Fatty Acids.

[32] Cullen J.C., H F.M., J L. (1975). The effect of dried mussel extract on an induced polyarthritis in rats. N. Z. Med. J..

[33] Singh M., Hodges L.D., Wright P.F.A., Cheah D.M.Y., Wynne P.M., Kalafatis N., Macrides T.A. (2008). The CO_2_-sfe crude lipid extract and the free fatty acid extract from *Perna canaliculus* have anti-inflammatory effects on adjuvant-induced arthritis in rats. Comp. Biochem. Physiol. B Biochem. Mol. Biol..

[34] Whitehouse M.W., Roberts M.S., Brooks R.M. (1999). Over the counter (otc) oral remedies for arthritis and rheumatism: How effective are they?. Inflammopharmacology.

[35] Mikami K., Hosokawa M. (2013). Biosynthetic pathway and health benefits of fucoxanthin, an algae-specific xanthophyll in brown seaweeds. Int. J. Mol. Sci..

[36] Rocha de Souza M.C., Marques C.T., Guerra Dore C.M., Ferreira da Silva F.R., Oliveira Rocha H.A., Leite E.L. (2007). Antioxidant activities of sulfated polysaccharides from brown and red seaweeds. J. Appl. Phycol..

[37] Lawson B.R., Belkowski S.M., Whitesides J.F., Davis P., Lawson J.W. (2007). Immunomodulation of murine collagen-induced arthritis by *N*,*N*-dimethylglycine and a preparation of *Perna canaliculus*. Evid.-Based Complement. Altern. Med..

[38] Mani S., Lawson J.W. (2006). *In vitro* modulation of inflammatory cytokine and igg levels by extracts of *Perna canaliculus*. BMC Complement. Altern. Med..

